# Role of *D-GADD45* in JNK-Dependent Apoptosis and Regeneration in *Drosophila*

**DOI:** 10.3390/genes10050378

**Published:** 2019-05-18

**Authors:** Carlos Camilleri-Robles, Florenci Serras, Montserrat Corominas

**Affiliations:** Departament de Genètica, Microbiologia i Estadística, Facultat de Biologia and Institut de Biomedicina (IBUB), Universitat de Barcelona, Barcelona 08028, Spain; carloscamilleri@ub.edu (C.C.-R.); fserras@ub.edu (F.S.)

**Keywords:** GADD45, JNK, p38, development, regeneration, imaginal discs

## Abstract

The GADD45 proteins are induced in response to stress and have been implicated in the regulation of several cellular functions, including DNA repair, cell cycle control, senescence, and apoptosis. In this study, we investigate the role of D-GADD45 during *Drosophila* development and regeneration of the wing imaginal discs. We find that higher expression of *D-GADD45* results in JNK-dependent apoptosis, while its temporary expression does not have harmful effects. Moreover, D-GADD45 is required for proper regeneration of wing imaginal discs. Our findings demonstrate that a tight regulation of *D-GADD45* levels is required for its correct function both, in development and during the stress response after cell death.

## 1. Introduction

The Growth Arrest and DNA Damage-inducible 45 (GADD45) family of proteins acts as stress sensors in response to various stimuli. The first *GADD45* gene was identified in mammals on the basis of its increased expression after growth cessation signals or treatment with DNA-damaging agents [1,2]. This gene, renamed as *GADD45α*, is a member of a highly conserved family, together with *GADD45β* and *GADD45γ*. *GADD45* genes encode for small (18 kd) and highly acidic proteins that can be both nuclear and cytoplasmic [3,4]. The expression of all *GADD45* genes is induced after exposure to several genotoxic or oxidative agents, such as ultraviolet radiation, methyl methanesulfonate (MMS) or hydrogen peroxide [1] and different family members have been implicated in a variety of responses to cell injury, including cell cycle checkpoints, apoptosis, and DNA repair (reviewed in [5,6]). GADD45 is also known to participate in the regeneration of zebrafish fin and retina [7,8,9].

Since GADD45 proteins do not have enzymatic properties, it has been suggested that they perform their functions by physically interacting with partner proteins (reviewed in [5]). Thus, upon stress induction, GADD45 interacts with different proteins involved in the different stress responses [5,10,11]. Although GADD45 proteins show complex regulation and numerous effectors, many of the prominent roles for the GADD45 proteins are associated with signaling mediated by mitogen-activated protein kinases (MAPK) [11,12,13,14,15,16]. This association is rather complex for c-Jun N-terminal kinase (JNK) and p38 proteins, members of the MAPKs pathways, which can contribute to GADD45 induction and at the same time are effectors of GADD45 signaling [11]**.** JNK and p38 pathways are also activated upon cellular stress, initiating the signaling modules that lead to the transcription of target genes involved in growth, differentiation, survival and apoptosis (reviewed in [17]). JNK and p38 can exert antagonistic effects on cell proliferation and survival, which depend on cell type-specific differences, as well as on the intensity and duration of the signal [18].

In mammals, GADD45 proteins directly bind to and activate MTK1/MEKK4, a MAP Kinase Kinase Kinase (MAP3K) upstream of JNK and p38 [12,16]. Other studies have revealed a putative interaction between GADD45ß, and ASK1, another MAP3K upstream of JNK and p38 [14]. However, it has been proposed that GADD45ß also interacts with MKK7, a MAP2K downstream of MTK1 and ASK1, inhibiting its kinase activity in mouse fibroblasts [14,15,19,20]. All these observations suggest that the effect of GADD45 on JNK signaling might be tissue-specific [21].

*D-GADD45* is the only member of the GADD45 family found in *Drosophila*, and it contains a single exon encoding a 163-amino acid protein. *D-GADD45* expression seems strongly dependent on the inflammatory response. *D-GADD45* was found to be upregulated upon activation of the immune response, but not following different stress stimuli including genotoxic stress [22]. By using a microarray screen to compare gene expression after laser wounding, *D-GADD45* was also identified as an inflammation-associated gene [23]. The effects of inducing the expression of *D-GADD45* seem to be tissue-specific also in the fly: overexpression of *D-GADD45* in the nervous system increases the lifespan of flies [24,25]. However, increased expression in somatic cells leads to apoptosis and in the germline causes several patterning and polarity defects [22]. *D-GADD45* was also found to be strongly upregulated in imaginal discs during regeneration. The expression of *D-GADD45* is rapidly increased upon damage, but after the initial steps of the stress response, when the tissue has still not completely regenerated, the expression of *D-GADD45* is recovered to the levels observed prior to damage [23,26,27]. This suggests a putative role of D-GADD45 only in the initial steps of regeneration. Moreover, damage also activates p38 and induces tolerable levels of JNK, which are essential for wound healing [28,29,30].

Here, we use *Drosophila* wing imaginal discs to study the role of *D-GADD45* during development and in the activation of the JNK signaling pathway. We found that a sustained activation of *D-GADD45* leads to JNK-dependent cell death, whereas transient expression of *D-GADD45* does not have detrimental effects. Moreover, the activation of *D-GADD45* also induces a decrease in proliferation, which is independent of the activation of the JNK signaling pathway. We also found that, while *D-GADD45* is dispensable during wing development under normal conditions, it becomes essential for regeneration. These findings suggest that *D-GADD45* could act as an in vivo stress sensor upstream of the MAP3K signaling cascade in *Drosophila*.

## 2. Materials and Methods

### 2.1. Drosophila Strains

The following Drosophila melanogaster strains were used: *TRE-DsRed.T4* [31], *ci-Gal4* (from R. Holmgren), *sal^E/Pv^-LHG* [30], *lexO-rpr* [30], *en-Gal4* (from G. Morata), and *UAS-GFP (RRID:BDSC_4776)*, *UAS-RNAi-Ask1 (RRID:BDSC_35331)*, *UAS-RNAi-Mekk1 (RRID:BDSC_35402)*, *UAS-bsk^DN^ (RRID:BDSC_9311)*, *UAS-D-GADD45 (RRID:BDSC_81038)*, *UAS-RNAi-D-GADD45 (RRID:BDSC_35023)* and *tubGal80^TS^ (RRID:BDSC_7017)* from the Bloomington Drosophila Stock Center. *Hh-Gal4* is described in FlyBase (https://flybase.org/).

### 2.2. Activation of Transgenes Using the Gal4/UAS System

The Gal4 and UAS lines used are indicated for each experiment. For sustained activation of transgenes, flies were kept at 25 °C until 96 h after egg laying, when they were dissected and stained. To study adult phenotypes, flies were kept at 25 °C until adulthood.

For transient experiments, the expression of *Gal4* was controlled by the thermo sensitive repressor *tubGal80^TS^*. Flies were kept at 17 °C until 192 h after egg laying (equivalent to 96 h at 25 °C) to prevent the expression of the constructs. They were subsequently kept at 29 °C for 6, 8 or 11 h. After that time, wing discs were immediately dissected and stained. To study the size and patterning of adult wings the vials were kept at 17 °C until adulthood.

### 2.3. Genetic Ablation and Dual Gal4/LexA Transactivation System

Cell death was induced as previously described [29,32]. We used the *sal^E/Pv^-LHG* driver [30], which contains the binding domain of *LexA* and the activator domain of *Gal4*, which is recognized by the inhibitor *Gal80^TS^*, to induce the expression of the pro-apoptotic gene *reaper* (*rpr*) cloned downstream of the *LexA* operator *LexO*. We simultaneously used *Gal4* to express either *UAS-D-GADD45* or *UAS-RNAi-D-GADD45* under the control of *ci-Gal4*. The system was controlled by the thermo sensitive *Gal4*-repressor *tubGal80^TS^*.

Embryos were kept at 17 °C until the 192 h after egg laying (equivalent to 96 h at 25 °C) to prevent *rpr* expression. They were subsequently moved to 29 °C for 11 h and then back to 17 °C until adulthood. Controls without *rpr* expression were always treated in parallel.

### 2.4. Immunohistochemistry and Statistics

Immunostaining was performed using standard protocols. Primary antibodies were P-Histone-H3 (rabbit 1:1000; Millipore, Burlington, MA, USA), Mmp-1 cocktail of three antibodies (3A6B4, 3B8D12 and 14A3D2, mouse 1:100 each; Developmental Studies Hybridoma Bank, Iowa, IA, USA), Cubitus interruptus (rat 1:25, Developmental Studies Hybridoma Bank) and P-JNK (rabbit 1:100; Promega, Madison, WI, USA).

Fluorescently labeled secondary antibodies were from Life Technologies (Carlsbad, CA, USA). Discs were mounted on SlowFade (Life Technologies). To label nuclei we used TO-PRO-3 (1:1000, Life Technologies), YO-PRO-1 (1:1000, Molecular Probes, Eugene, OR, USA) or Sytox Orange (1:10.000, Molecular Probes).

For apoptotic cell detection, we used the terminal deoxynucleotidyl transferase dUTP nick end labeling (TUNEL) assay, for which we used the fluorescently labelled Alexa Fluor 647-aha-dUTP (Thermo Fisher Scientific, Waltham, MA, USA) and incorporated using terminal deoxynucleotidyl transferase (Roche Diagnostics, Mannheim, Germany).

The apoptotic index was calculated after manually counting the number of cells positive for TUNEL in the anterior and posterior compartments of the disc, for all genotypes shown. The apoptotic index for each compartment was calculated as the fraction of apoptotic cells in that compartment multiplied by 100. Error bars indicate standard deviation of the mean. To compare apoptotic indexes, we used two-way analysis of variance (ANOVA) followed by Tukey post-hoc test on variables transformed to logarithmic scale.

The mitotic index was calculated after manually counting the number of P-Histone-H3 (PH3) positive cells in the anterior and posterior compartments, for all genotypes shown. The mitotic index for each compartment was calculated as the fraction of mitotic cells in that compartment multiplied by 100. Error bars indicate standard deviation of the mean. To compare mitotic indexes, we used two-way ANOVA followed by Tukey post-hoc test.

### 2.5. Test for Adult Wing Phenotypes

Female adult flies were fixed in glycerol:ethanol (1:2) for 24 h. Wings were dissected on water and then washed with ethanol. Then they were mounted on 6:5 lactic acid:ethanol and analyzed and imaged under a microscope. We considered wings as aberrant when missing veins or crossveins and/or when notches were present in the wing blade. Wing size was considered as the area inside the wing blade perimeter, as represented in Results section hh>GFP image.

Wing size and phenotype for each genotype is represented as a boxplot. Boxes represent the 1st, 2nd and 3rd quartiles. Whiskers represent interquartile range.

## 3. Results

### 3.1. Sustained Activation of D-GADD45 Induces JNK-Dependent Apoptosis

**Figure 1:**
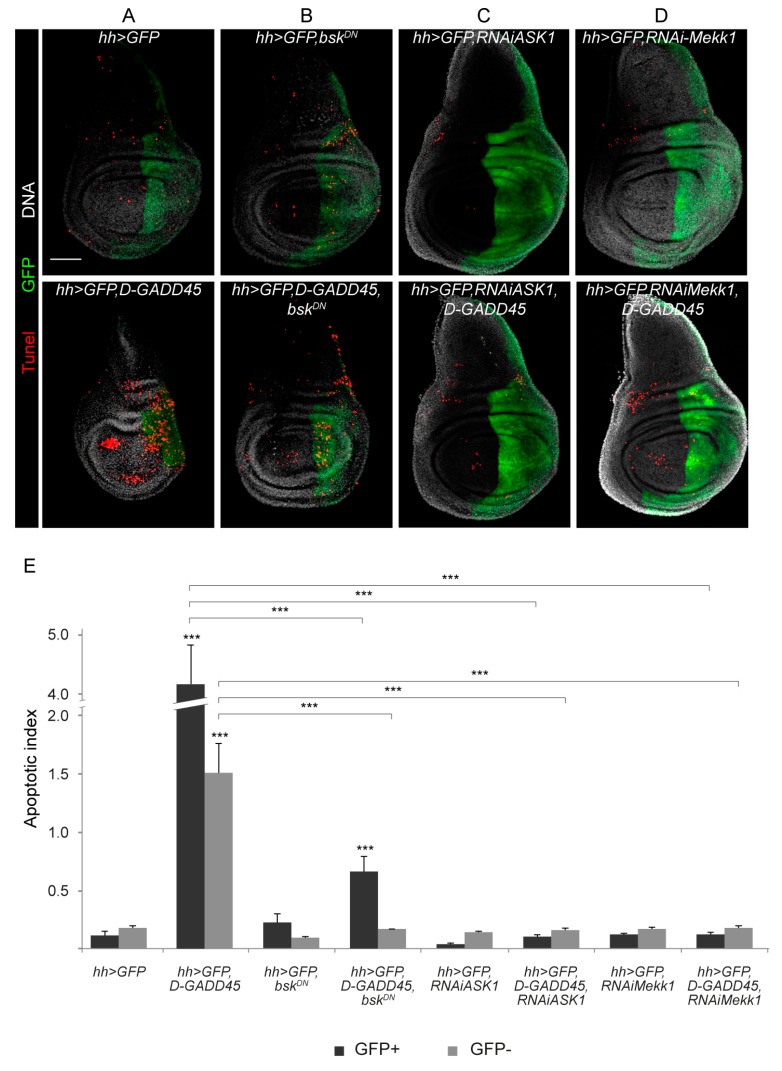
Sustained expression of *D-GADD45* induces JNK-dependent cell death. (**A**–**D**) TUNEL assay of wing discs labeling apoptotic cells after sustained expression of each construct in the posterior (*hedgehog*, *hh*) compartment. Apoptotic cells (red), posterior compartment (green) and DNA (white). Scale bar: 50 μm. (**E**) Histogram showing the apoptotic index for each genotype in the green fluorescent protein (GFP)-positive and GFP-negative compartments. Error bars indicate standard deviation. *N* ≥ 4 discs for each genotype. **p < 0.01. ***p < 0.001.

To get insight into the relationship between *GADD45* levels and their putative role, we first determined the effect of increased expression of *D-GADD45* in wing imaginal discs by employing the Gal4/UAS system [33]. For this, we used *UAS-D-GADD45* under the control of *hedgehog* (*hh)-Gal4*, which is expressed in the posterior compartment of the imaginal discs, allowing us to compare the effects on the posterior (autonomous) and anterior (non-autonomous) compartments within the same disc. We activated the Gal4/UAS system throughout development until the third instar larval (L3) stage, when wing discs were dissected. We found a reduction in the whole disc size, including a reduction in size of the posterior compartment (Figure 1A). We also measured the apoptotic index in the posterior (GFP-positive) and anterior (GFP-negative) compartments and we observed a significant increase in the apoptotic index of both compartments when *D-GADD45* was upregulated compared to control discs (Figure 1A).

As mentioned before, it is well known that mammalian GADD45 proteins are able to interact to and activate the JNK signaling pathway [12,16]. Since sustained activation of the JNK cascade also has proapoptotic effects [34,35], we next checked whether sustained expression of *D-GADD45* activates the JNK pathway. We observed a clear increase in the activity of JNK upon increased *D-GADD45* expression, which indicates that D-GADD45 can activate JNK in the wing imaginal discs (Figure S1). To assess whether the increase in apoptosis is JNK dependent, we combined the expression of *D-GADD45* and the expression of a dominant negative form of the serine/threonine-protein kinase Basket (Bsk), a key downstream component of the JNK cascade, to inhibit the pathway. These discs showed a significant reduction of the apoptotic index in both compartments compared to discs expressing only *D-GADD45* (Figure 1A–B).

To gain further insight into the interaction between D-GADD45 and the JNK pathway, we examined the genetic interaction between two different MAP3Ks involved in the JNK pathway: the *Drosophila* ortholog of mammalian MTK1, Mekk1, and Ask1. In these experiments we combined the expression of *D-GADD45* and the expression of RNA interference (RNAi) constructs against either Mekk1 or Ask1. Similar to observations made when inhibiting the JNK pathway using the dominant negative form of Basket, depletion of Mekk1 or Ask1 significantly reduced the apoptosis caused by D-GADD45 (Figure 1C–D).

**Figure 2:**
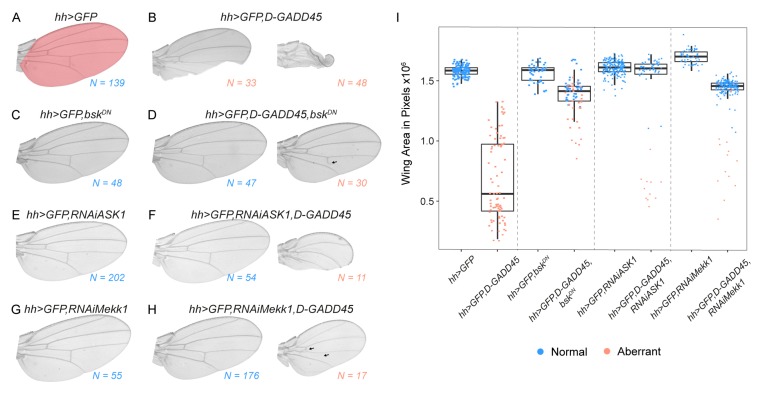
Sustained expression of D-GADD45 results in smaller and aberrant wings. (**A**–**H**) Adult wings showing the predominant phenotypes observed after sustained expression of each construct in the posterior (*hedgehog*, *hh*) compartment. Number of wings analyzed is indicated for each condition. Colored region in (**A**) represents the area selected to calculate wing size. (**I**) Box plot showing the average area of adult wings obtained after the expression of each combination of constructs in the posterior (*hedgehog*, *hh*) compartment. Each dot represents one wing; wild-type pattern (blue) and aberrant pattern (orange).

We also scored the adult wing phenotypes of flies in which *D-GADD45* was overexpressed in the posterior compartment and found aberrant and smaller size wings compared to controls (Figure 2A–B). Consistent with the phenotype observed in the imaginal discs, the inhibition of the JNK cascade, either by expressing the mutant form of *basket* or by depleting *Mekk1* or *Ask1* by RNAi, was sufficient to rescue the phenotypes, resulting in normal-sized and well-patterned wings (Figure 2C–H).

**Figure 3:**
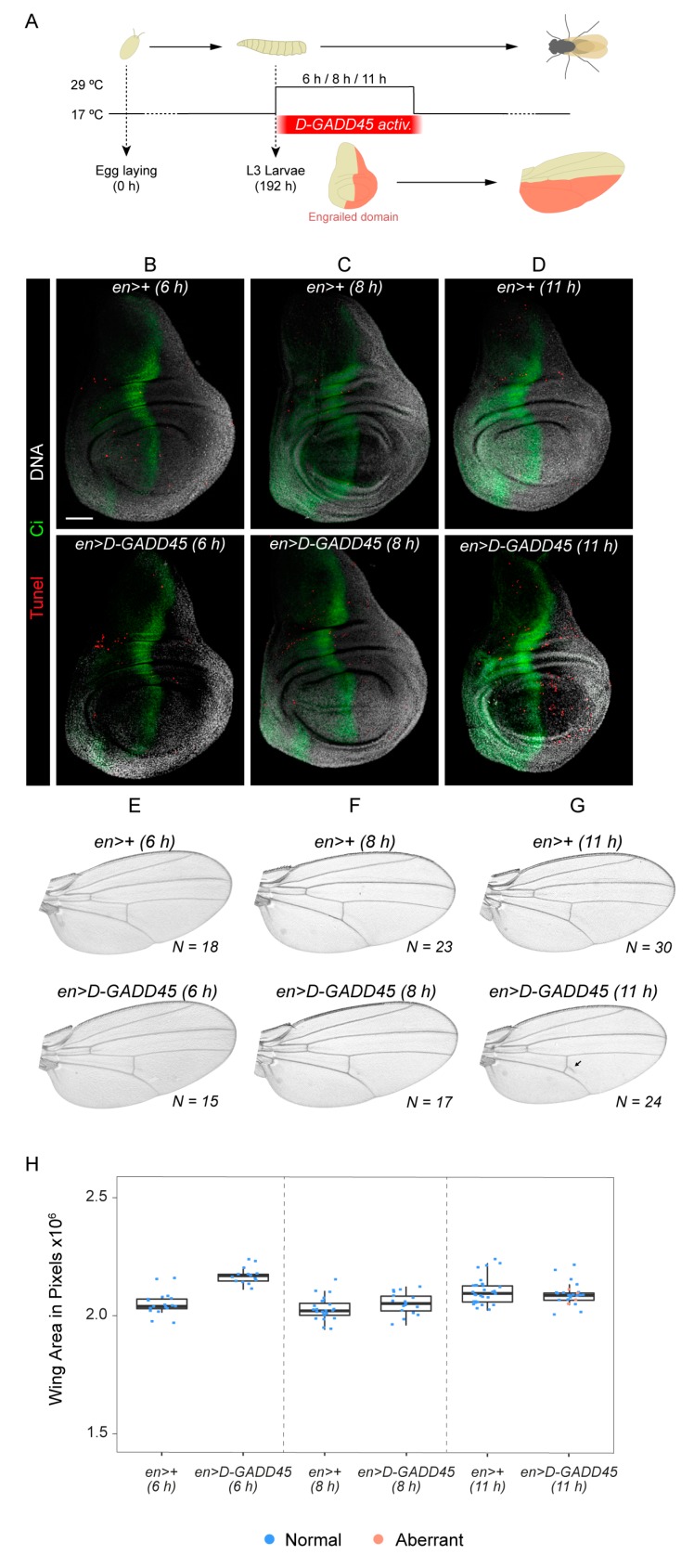
Transient expression of *D-GADD45* is not sufficient to induce cell death. (**A**) Schematic representation of *D-GADD45* transient activation in the posterior (*engrailed, en*) compartment. The affected domain is represented in orange. (**B**–**D**) TUNEL assay of wing imaginal discs labeling apoptotic cells (red), the anterior compartment (Ci: Cubitus interruptus, green) and DNA (white). *N* = 5 for each condition. Scale bar: 50μm. (**E**–**G**) Adult wings showing the predominant phenotype observed after transient expression of each construct in the posterior (*engrailed*, *en*) compartment. Number of wings analyzed is indicated for each condition. (**H**) Box plots showing the average area of adult wings. Each dot represents one wing; normal (blue) and aberrant pattern (orange).

Since sustained expression of *D-GADD45* induces JNK-dependent cell death, we analyzed whether transient expression of *D-GADD45* is sufficient to induce apoptosis. To do so, we transiently activated the expression of *D-GADD45* in the posterior compartment of the wing disc (Figure 3A). After 6 and 8 h of *D-GADD45* activation, no differences were observed compared to control discs (Figure 3B–C). However, after 11 h of induction there was an increase in the number of apoptotic cells in the posterior compartment (Figure 3D). No wing patterning defects were observed after 6 or 8 h (Figure 3E–F), and minor vein alterations were detected after 11 h (Figure 3G). A slight increase in wing size was observed after 6 h of *D-GADD45* induction (Figure 3H).

Altogether, these results demonstrate that the detrimental effects of *D-GADD45* are due to the exposure to sustained levels of expression and that transient expression of *D-GADD45* is not sufficient to induce cell death, since we could not detect apoptosis after 6 and 8 h of induction. Our findings also suggest that D-GADD45 interaction with the JNK pathway could be mediated at the MAP3K level, by Mekk1 and Ask1 kinases.

### 3.2. Increased Expression of D-GADD45 Decreases Cell Proliferation Independently of the JNK Signaling Pathway

**Figure 4:**
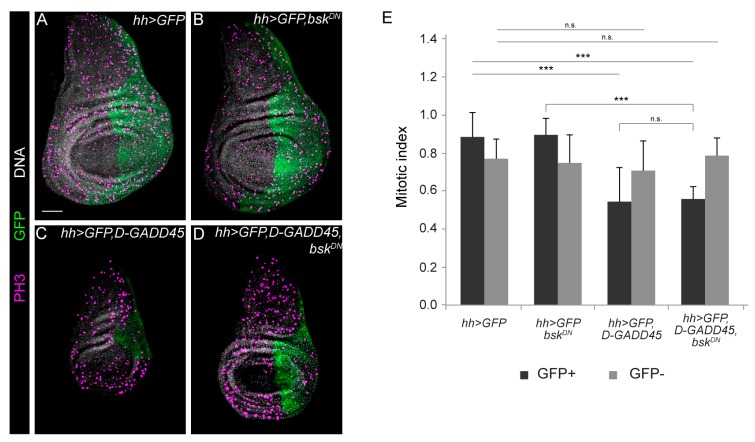
Sustained expression of *D-GADD45* decreases cell proliferation independently of JNK activation. (**A**–**D**) Immunostaining of wing discs with P-histone-H3 labeling mitosis (magenta), the posterior compartment (green) and DNA (white). Scale bar: 50 μm. (**E**) Histogram showing the mitotic index of PH3 labeling for each genotype in the GFP-positive and GFP-negative compartments. *N* ≥ 10 for genotype. ***p < 0.001.

Overexpression of GADD45 results in cell growth suppression in numerous mammalian cell lines [3,36,37,38]. Therefore, we analyzed whether higher levels of *D-GADD45* would also affect proliferation in the imaginal discs. For this, we induced *D-GADD45* expression in the posterior compartment of the wing discs and calculated the mitotic index using P-histone-H3 (histone H3 phosphorylated) as a mitotic marker. We found that increased *D-GADD45* expression in the posterior compartment reduced the whole disc size (Figure 1A and 4C) and significantly reduced mitosis in this compartment (Figure 4C). The mitotic index in the anterior compartment was, however, comparable to that in control discs (Figure 4A).

We next inhibited the JNK cascade by expressing the dominant negative form of *basket*, preventing *D-GADD45*-induced apoptosis. We found that, in these discs, mitosis in the anterior compartment was also equivalent to that in control discs (Figure 4B–D); however, proliferation in the posterior compartment was still significantly reduced but did not differ significantly from that found in discs in which only *D-GADD45* was activated (Figure 4C–D). Altogether, these results suggest that besides apoptosis, *D-GADD45* induces an autonomous decrease in proliferation independently of the activation of the JNK cascade.

### 3.3. D-GADD45 is Required for Regeneration of Wing Imaginal Discs

**Figure 5:**
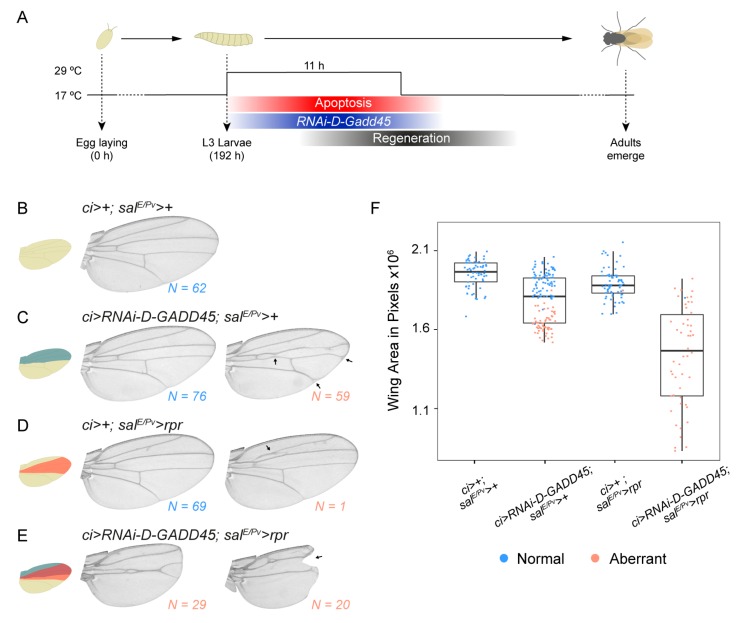
Depletion of *D-GADD45* severely impairs wing regeneration. (**A**) Schematic representation of the regeneration model used. (**B**–**E**) Adult wings showing the predominant phenotypes observed in each condition. Number of wings analyzed is indicated for each condition. (**F**) Box plot showing the average area of adult wings after 11 h of induction. Each dot represents one wing; wild-type pattern (blue) and aberrant pattern (orange).

Because we had previously observed that *D-GADD45* was transiently upregulated following physical injury or genetic induction of cell death [26,27], we analyzed the ability of *D-GADD45*-depleted discs to regenerate. We scored wing regeneration after the induction of cell death by expressing the proapoptotic gene *reaper* (*sal^E/Pv^* > *rpr*) while depleting *D-GADD45* by RNAi in the anterior compartment using the driver *cubitus interruptus* (*ci* > *RNAi-D-GADD45*) (Figure 5A). While control wings were normal in size and pattern, transient depletion of *D-GADD45* by RNAi produced visible defects in wing size and patterning in around half of the analyzed wings (Figure 5B–C). In regenerating animals, depletion of *D-GADD45* completely impaired regeneration, resulting in aberrant wings lacking the pattern and size of controls (Figure 5D–F). The presence of notches in the wing blade also indicated that the missing tissue had not been recovered, suggesting that activation of *D-GADD45* is required for wing repair after cell death. These findings show that *D-GADD45* contributes to regeneration.

## 4. Discussion

In this work, we describe a role for the stress sensor *D-GADD45* in *Drosophila*. Our results, together with previous observations, indicate that the levels of *D-GADD45* must be controlled both, during normal development as well as after the induction of cell death.

We also reveal the relationship between the D-GADD45 protein and the JNK signaling pathway during development. A previous study in flies already showed a genetic interaction in the germline between D-GADD45 and MAP Kinase Kinases (Hemipterous and Licorne), members of the JNK and p38 signaling pathways, respectively [22]. Our data show a genetic interaction of D-GADD45 and the JNK signaling pathway at the level of MAP3Ks. In mammals, binding of GADD45 to MTK1, ortholog of *Drosophila* Mekk1, leads to the auto-phosphorylation of its kinase domain, allowing MTK1 to trigger the JNK signaling cascade [12,16]. Previous studies have also revealed physical binding between ASK1 and GADD45 in human cells, although this interaction was thought to be non-functional [14]. Further analyses are required to uncover whether the molecular mechanism of GADD45-mediated activation of the JNK pathway is conserved in *Drosophila*.

In mammals, overexpression of GADD45 has been found to induce G2/M phase arrest in numerous cell lines [3,36,37,39,40]. Here we show that a sustained increase of *D-GADD45* levels results in an increase in apoptosis and a decrease in cell proliferation. Although several studies have implicated JNK signaling in G2/M phase arrest [41,42], we still observed a decrease in mitosis when activating *D-GADD45* and inhibiting the JNK cascade through development, suggesting that the activation of JNK is dispensable in *Drosophila* for the *D-GADD45*-mediated effect in cell proliferation. On the other hand, it has been described that the *Drosophila* wing size is regulated by JNK signaling during development [43]. Thus, we cannot discard that a short induction of *D-GADD45* (6h) may activate a JNK-mediated proliferative response.

One of the early responses to damage in the wing imaginal discs is the activation of the JNK signaling cascade, which is required for regeneration [28,29,44]. In this system, induction of cell death activates p38 and induces tolerable levels of JNK, which are essential for wound healing [30]. Moreover, we have recently demonstrated the requirement for Ask1 during wing regeneration [45]. We hypothesize here that D-GADD45 could be required to regulate the activity of JNK by activating Ask1 and Mekk1. We cannot rule out the possibility, however, that other members of the JNK signaling pathway could also interact with D-GADD45 during the stress response in *Drosophila*, as observed in other systems [14,15,19,20]. Similar to mammals [11]**,** it is likely that members of the MAPK family that are effectors of GADD45 signaling, contribute, at the same time to GADD45 induction.

Finally, it is tempting to speculate about the possible mechanisms behind the tight regulation of *D-GADD45* levels during regeneration. On the one hand, *D-GADD45* could be a direct downstream target of the JNK pathway. Both, p38 and JNK are required only during the early response [30] and the expression of *D-GADD45* shows an increase/decrease pattern during regeneration [26,27]. In addition, the promoter region of *D-GADD45* contains putative binding sites for the AP1 (activator protein-1) protein, the transcription factor downstream of the stress-responding JNK pathway [26]. On the other hand, a degradation of *D-GADD45* messenger RNA (mRNA) by nonsense-mediated decay (NMD) has been described as essential for viability in flies [46]. This observation suggests that when *D-GADD45* mRNA levels reach a certain threshold, the NMD pathway would destroy these transcripts, reducing the amount of the D-GADD45 protein to the appropriate levels to prevent D-GADD45-mediated apoptosis.

## Supplementary Materials

The following are available online at https://www.mdpi.com/2073-4425/10/5/378/s1, Figure S1: Sustained expression of *D-GADD45* activates JNK.
