# Supplementary material for: Role of D-GADD45 in JNK-Dependent Apoptosis and Regeneration in Drosophila
Source: Genes (Basel). 2019 May 18;10(5):378. doi: 10.3390/genes10050378 (PMC6562583; doi:10.3390/genes10050378)
Supplement: Supplementary file 1 [file genes-10-00378-s001.pdf]

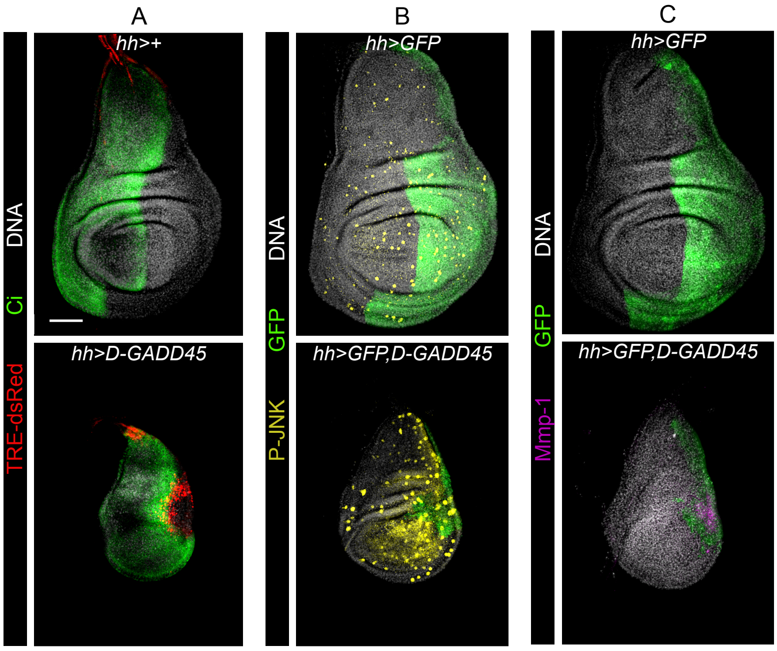

Figure S1. Sustained expression of *D-GADD45* activates JNK. Detection of JNK activity using the reporter line TRE-dsRed (A) and two different markers, P-JNK (B) and Mmp-1 (C). Green: anterior compartment (A), posterior compartment (B-C), white: DNA, red: TRE-dsRed, yellow: P-JNK and magenta: Mmp-1. Bar: 50µm. N ≥ 5 for each genotype
